# Muscle-specific inflammation induced by MCP-1 overexpression does not affect whole-body insulin sensitivity in mice

**DOI:** 10.1007/s00125-015-3822-2

**Published:** 2015-12-12

**Authors:** Inkie J. A. Evers-van Gogh, Antwi-Boasiako Oteng, Sheril Alex, Nicole Hamers, Milene Catoire, Rinke Stienstra, Eric Kalkhoven, Sander Kersten

**Affiliations:** Molecular Cancer Research and Center for Molecular Medicine, University Medical Centre Utrecht, Utrecht, the Netherlands; Nutrition, Metabolism and Genomics Group, Division of Human Nutrition, Wageningen University, Stippeneng 4, 6708 WE Wageningen, the Netherlands

**Keywords:** Inflammation, Insulin resistance, MCP-1, Muscle-specific, Myokine, Obesity, Type 2 diabetes

## Abstract

**Aims/hypothesis:**

Obesity is associated with a state of chronic low-grade inflammation that is believed to contribute to the development of skeletal muscle insulin resistance. However, the extent to which local and systemic elevation of cytokines, such as monocyte chemoattractant protein 1 (MCP-1), interferes with the action of insulin and promotes insulin resistance and glucose intolerance in muscle remains unclear. Here, we aim to investigate the effect of muscle-specific overexpression of MCP-1 on insulin sensitivity and glucose tolerance in lean and obese mice.

**Methods:**

We used *Mck*–*Mcp-1* transgenic (Tg) mice characterised by muscle-specific overexpression of *Mcp-1* (also known as *Ccl2*) and elevated plasma MCP-1 levels. Mice were fed either chow or high-fat diet for 10 weeks. Numerous metabolic variables were measured, including glucose and insulin tolerance tests, muscle insulin signalling and plasma NEFA, triacylglycerol, cholesterol, glucose and insulin.

**Results:**

Despite clearly promoting skeletal muscle inflammation, muscle-specific overexpression of *Mcp-*1 did not influence glucose tolerance or insulin sensitivity in either lean chow-fed or diet-induced obese mice. In addition, plasma NEFA, triacylglycerol, cholesterol, glucose and insulin were not affected by MCP-1 overexpression. Finally, in vivo insulin-induced Akt phosphorylation in skeletal muscle did not differ between *Mcp-1*-Tg and wild-type mice.

**Conclusions/interpretation:**

We show that increased MCP-1 production in skeletal muscle and concomitant elevated MCP-1 levels in plasma promote inflammation in skeletal muscle but do not influence insulin signalling and have no effect on insulin resistance and glucose tolerance in lean and obese mice. Overall, our data argue against MCP-1 promoting insulin resistance in skeletal muscle and raise questions about the impact of inflammation on insulin sensitivity in muscle.

**Electronic supplementary material:**

The online version of this article (doi:10.1007/s00125-015-3822-2) contains peer-reviewed but unedited supplementary material, which is available to authorised users.

## Introduction

Over recent decades, the global prevalence of obesity has been increasing steadily, concurrent with major changes in diet and lifestyle. Obesity is often accompanied by a number of metabolic perturbations, including insulin resistance, hyperinsulinaemia, dyslipidaemia and hypertension, which together substantially increase the risk for type 2 diabetes and cardiovascular disease [1–4]. Development of insulin resistance is generally considered as an early event that drives many of the metabolic disturbances associated with obesity. Since insulin resistance was first recognised several decades ago, a number of ideas have been put forward to try and explain the link between excess adiposity and reduced tissue sensitivity to insulin. Currently, major emphasis is placed on the detrimental effect of fat stored outside the adipose compartment. Additionally and alternatively, inflammation is increasingly believed to play a pivotal role in the development of insulin resistance and type 2 diabetes (reviewed in [5]). According to this so-called inflammatory hypothesis, obesity is characterised by expansion of the adipose depot, giving rise to adipocyte hypertrophy, hypoxia and endoplasmic reticulum (ER) stress, which in turn causes enhanced production of cytokines and chemokines [6–8]. Cytokines such as TNFα, IL-1β, IL-6, IL-8 and monocyte chemoattractant protein 1 (MCP-1) promote the attraction of different immune cells in obese adipose tissue, including proinflammatory macrophages and B cells, whereas regulatory T cells, natural killer T cells and eosinophils become less abundant [9]. These immune cells are producers of a host of different cytokines/chemokines and together with the hypertrophic adipocytes contribute to a state of chronic low-grade inflammation. In vitro studies abound indicating that proinflammatory cytokines are able to inhibit insulin signalling and thus promote the development of insulin resistance in fat cells and muscle cells. For example, Eckel and colleagues found that the chemokine MCP-1 impaired insulin signalling and reduced insulin-stimulated glucose uptake in muscle cells, suggesting that MCP-1 may represent a molecular link in the negative crosstalk between adipose tissue and skeletal muscle [10]. What is less clear is whether the obesity-induced adipose tissue inflammation leads to the increments in cytokine levels in blood plasma necessary to interfere with the action of insulin and to cause insulin resistance in muscle in vivo. This is particularly relevant since it has been calculated that approximately 80% of insulin-induced glucose disposal is accounted for by skeletal muscle glucose uptake. Studies performed using mice characterised by fat-specific overexpression of *Mcp-1* (also known as *Ccl2*) or expression of a dominant-negative mutant of *Mcp-1* suggest that fat-derived MCP-1 reduces glucose tolerance and insulin sensitivity and blunts insulin-mediated signalling [11, 12]. However, the full scope of the role of adipose tissue inflammation and the impact of specific cytokines such as MCP-1 on whole-body glucose tolerance and insulin’s action in skeletal muscle remains ambiguous.

Importantly, recent studies indicate that elevated MCP-1 expression not only occurs during obesity but is also a feature of physical exercise. Specifically, levels of *MCP-1* mRNA in skeletal muscle and levels of MCP-1 protein in plasma were found to be increased upon acute exercise, leading to the classification of MCP-1 as myokine or exercise factor [13–17]. In skeletal muscle, MCP-1 is known to promote macrophage infiltration after (severe) tissue damage [18, 19]. In contrast, chronic exercise results in a decrease in MCP-1 plasma levels [20, 21]. The features of MCP-1 are reminiscent of IL-6. Production of IL-6 in adipose tissue has been shown to be increased during obesity and contributes to obesity-associated low-grade inflammation (reviewed in [22]). At the same time, production of IL-6 in skeletal muscle and plasma IL-6 levels are increased in response to acute exercise and have been suggested to potentially confer a positive effect on insulin signalling and insulin sensitivity in skeletal muscle [23], although recent data argue against that notion [24]. How inflammatory mediators may have a beneficial role in the context of physical exercise yet promote insulin resistance during obesity represents somewhat of a conundrum.

The aim of the present study was to assess the extent to which elevation of MCP-1 production in muscle and elevated MCP-1 levels in plasma may be able to interfere with insulin signalling in skeletal muscle and promote insulin resistance. To this end, we took advantage of *Mck*–*Mcp*-*1* transgenic (Tg) mice, which specifically overexpress *Mcp-1* in skeletal muscle. Our studies indicate that elevation of MCP-1 production in skeletal muscle and elevated MCP-1 levels in plasma promote inflammation in skeletal muscle but do not influence insulin signalling in skeletal muscle and have no effect on insulin resistance and glucose tolerance in lean and obese mice.

## Methods

### Animals

Animal studies were carried out using wild-type (WT) and *Mck–Mcp-1* Tg mice, all on a C57Bl/6J background [25]. The mice were kindly donated by D. Patsouris (Université Claude Bernard Lyon 1, France). Only male mice were used. Mice were kept in temperature controlled rooms (21 ± 1°C) on a 12 h light–dark cycle. For the studies using chow-fed mice, 16 WT and 19 *Mcp-1*-Tg mice were fed regular chow (RMH-B Arie Blok, Woerden, the Netherlands). At 14–17 weeks of age, eight mice per group were subjected to an intraperitoneal glucose tolerance test and a week later to an intraperitoneal insulin tolerance test. Mice were euthanised at 17–20 weeks of age after a 6 h fast at zeitgeber time (ZT)7–ZT9. Before being euthanised, eight mice of each genotype received an intraperitoneal injection of insulin (2 U/kg body weight in 100 μl saline [154 mmol/l NaCl], *n* = 4 per genotype) or saline (100 μl, *n* = 4 per genotype) followed by euthanasia exactly 5 min later by cervical dislocation. For the remaining mice, blood was collected by orbital puncture under isoflurane anaesthesia and mice were euthanised by cervical dislocation. For the high-fat feeding study, 12 WT and 12 *Mcp-1*-Tg mice were fed regular chow (RMH-B Arie Blok) after weaning. At 12–16 weeks of age, mice were switched to a high-fat diet (HFD) containing 60 energy% fat (D12492; Research Diets Inc., New Brunswick, NJ, USA). Food intake and body weights were registered weekly. After 8 weeks of high-fat feeding, mice were subjected to an intraperitoneal glucose tolerance test and a week later to an intraperitoneal insulin tolerance test. Mice were euthanised after 10 weeks of high-fat feeding after a 6 h fast at ZT7–ZT9. Before being euthanised, four mice of each genotype received an intraperitoneal injection of insulin (2 U/kg body weight in 100 μl saline, *n* = 2 per genotype) or saline (100 μl, *n* = 2 per genotype) followed by euthanasia exactly 5 min later by cervical dislocation. For the remainder of the mice, blood was collected by orbital puncture under isoflurane anaesthesia and mice were euthanised by cervical dislocation. Tissues were excised and immediately frozen in liquid nitrogen followed by storage at −80°C. All experiments were blind to group and outcome assessment. All animal experiments were authorised by the animal welfare committee of Wageningen University.

### Intraperitoneal glucose tolerance test

After a 6 h fast mice were injected intraperitoneally with glucose (1.2 g/kg body weight for mice on chow, 0.8 g/kg body weight for mice on HFD). Blood was collected by tail bleeding after 0, 15, 30, 60, 90 and 120 min, and glucose was measured using an Accu-Chek compact (Roche Diagnostics, Almere, the Netherlands).

### Intraperitoneal insulin tolerance test

After a 6 h fast mice were injected intraperitoneally with insulin (0.75 U/kg body weight). Blood was collected by tail bleeding after 0, 15, 30, 45, 60 and 90 min, and glucose was measured using an Accu-Chek compact (Roche Diagnostics).

### Histological stainings

Histological analysis of musculus gastrocnemius morphology and macrophage infiltration was done using hematoxylin and eosin (H&E) staining. On the day of euthanasia, samples were frozen in isopentane mixed with dry ice and stored at −80°C. Tissue was embedded in Tissue-Tek O.C.T. Compound (Sakura Finetek, Alpen aan de Rijn, the Netherlands), sectioned at 10 μm in a cryostat chamber, fixed on a Superfrost glassslide, left to dry for 30 min and stored at −20°C. Samples were stained at room temperature in Mayer hematoxylin solution for 10 min and in eosin Y solution for 30 s.

### Measurement of plasma variables

Blood was collected into EDTA-coated tubes. Blood samples were placed on ice and centrifuged at 4°C for 10 min at 10,000 *g*. Plasma was collected and stored at −80°C. To measure plasma MCP-1 concentration a DuoSet ELISA Development kit against mouse MCP-1 was used (R&D Systems, Minneapolis, MN, USA). For insulin measurements an ultra-sensitive mouse insulin ELISA kit was used (Crystal chemicals, Downers Grove, IL, USA). For the other plasma measurements the following kits were used: NEFA Reagent set, Triglycerides Liquicolor, Cholesterol Liquicolor (all Instruchemie, Delfzijl, the Netherlands) and Glucose GOD FS (DiaSys, Holzheim, Germany). All measurements were performed according to the manufacturers’ protocols.

### Affymetrix microarray

Microarray analysis was performed on RNA of the musculus gastrocnemius as described in the electronic supplementary material (ESM) Methods.

### RNA isolation and quantitative real-time PCR

Total RNA was extracted from different mouse tissues using TRIzol reagent (Invitrogen, Bleiswijk, the Netherlands) and a Tissuelyser II (Qiagen, Venlo, the Netherlands). Reverse transcription was performed using Superscript II and oligo(dT) primers (Invitrogen). PCR-amplifications were carried out using iQ SYBR Green Supermix on a CFX384 Touch real-time PCR detection system (Bio-Rad, Hercules, CA, USA).

The sequences of the primers used for quantitative real-time PCR (qPCR) are shown in ESM Table 1. All genes were normalised to *36b4*. Primer efficiencies were determined using LinRegPCR v11.1 (http://LinRegPCR.nl) [26]. Relative expression of the transcript levels was calculated as described previously [27].

### Western blotting

Total protein extracts were obtained from musculus gastrocnemius to detect MCP-1. Tissue was homogenised using a Tissuelyser II (Qiagen) in protein extraction buffer without bromophenol blue (62.5 mmol/l Tris HCl, pH 6.8, 2% SDS, 10% glycerol and 10 mmol/l dithiothreitol) and protein concentration was measured using Bradford protein assay. Protein (50 μg) was boiled, subjected to SDS-PAGE and proteins transferred to nitrocellulose membrane (Immobilon; Millipore, Billerica, MA, USA). Membranes were blocked and incubated with anti-MCP-1 (R17; Santa Cruz, Dallas, TX, USA) and anti-Actin (Sigma Aldrich, St Louis, MO, USA).

To obtain total protein extracts from musculus gastrocnemius to detect phospho-Akt, tissue was homogenised using a Tissuelyser II (Qiagen) in protein extraction buffer with phosphatase inhibitors (50 mmol/l Tris HCl, pH 4.7, 1 mmol/l EDTA, 150 mmol/l NaCl, 1% NP40, 0.25% sodium deoxycholate, 2 mmol/l sodium orthovanadate and 5 mmol/l sodium fluoride, containing freshly added protease inhibitor cocktail [Roche Applied Science, Penzberg, Germany]). After being centrifuged at 20,800 *g* for 10 min at 4°C, supernatant fractions were collected and 50 μg of protein was boiled with Laemmli sample buffer. The protein was subjected to SDS-PAGE and transferred to a polyvinylidene difluoride membrane (Immobilon; Millipore). Membranes were blocked and incubated with anti-Akt (in house), anti-phospho-Akt (Ser473) (Cell Signaling Technology, Danvers, MA, USA) and anti-Actin (Sigma Aldrich). Quantification was carried out using ImageJ 1.49m (http://imagej.nih.gov).

### Statistical analysis

For statistical analysis Student’s *t* test was used. Data are means ± SEM and *p* < 0.05 is considered statistically significant. One mouse from the WT group, fed HFD, was excluded from subsequent analysis because it was mistakenly injected with a higher dose of insulin during the insulin tolerance test. Some qPCR data on gene expression were excluded from the results section because the genes were expressed at a low level, with C_t_ values greater than 32 in both the WT and *Mcp-1-*Tg mice.

## Results

### Skeletal muscle-specific overexpression of MCP-1 results in a local inflammatory response

**Fig. 1:**
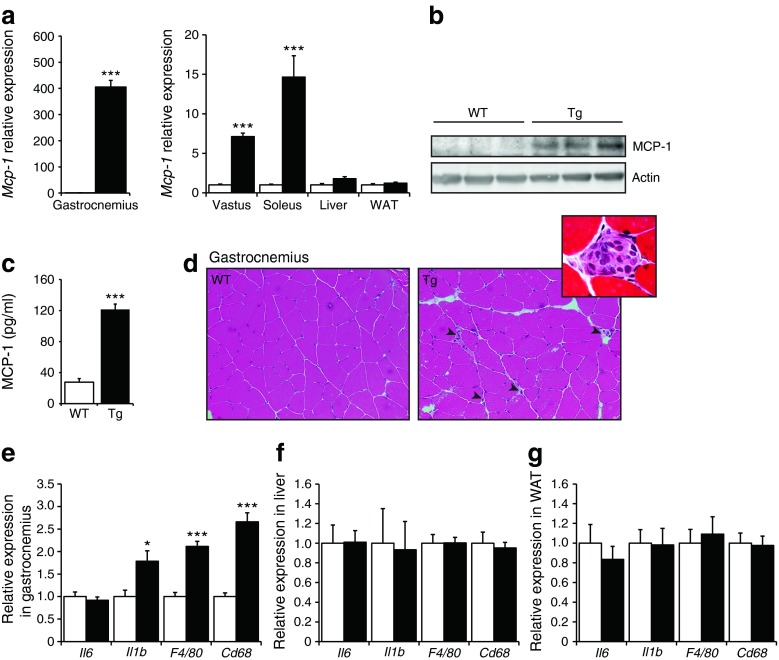
Skeletal muscle-specific overexpression of MCP-1 provokes inflammation. (**a**) Relative gene expression of *Mcp-1* measured by qPCR; *n* = 8 (WT mice) or *n* = 11 (*Mcp-1*-Tg mice). (**b**) Representative western blot analysis of MCP-1 overexpression in *Mcp-1*-Tg mice. (**c**) Plasma concentration of MCP-1; *n* = 8 (WT mice) or *n* = 11 (*Mcp-1*-Tg mice). (**d**) H&E staining of representative sections of musculus gastrocnemius of WT and *Mcp-1*-Tg mice (magnification × 200). Arrowheads indicate macrophage infiltration. Insert: H&E staining of musculus vastus (magnification ×1,000), showing immune cell infiltrate. **e**–**g**: Relative gene expression of *Il6*, *Il1b*, *F4/80* and *Cd68* in gastrocnemius (**e**), liver (**f**) and WAT (**g**) measured by qPCR; *n* = 8 (WT mice) or *n* = 11 (*Mcp-1*-Tg mice). White bars, WT mice; black bars, *Mcp-1*-Tg mice. Data are means ± SEM; *p < 0.05 and ***p < 0.001 according to Student’s *t* test for WT vs *Mcp-1*-Tg mice

To assess the impact of muscle-specific overexpression of MCP-1 on whole-body glucose homeostasis and insulin sensitivity, we used Tg mice that overexpress *Mcp-1* under the control of the *Mck* promotor [25]. The marked overexpression of *Mcp*-*1* in the transgenic mice was confirmed at the mRNA level in the musculus gastrocnemius, vastus and soleus, whereas *Mcp-1* was only weakly induced or not induced in the liver and white adipose tissue (WAT), respectively (Fig. 1a). Since the overexpression of *Mcp-1* was most pronounced in the gastrocnemius muscle, we used the gastrocnemius in all follow-up experiments. Overexpression of *Mcp-1* in the gastrocnemius of the *Mcp-1*-Tg mice was confirmed at the protein level (Fig. 1b). Furthermore, the plasma concentration of MCP-1 in the *Mcp-1*-Tg mice was increased approximately 4.4-fold compared with WT mice, indicating that MCP-1 is secreted from skeletal muscle (Fig. 1c). H&E staining indicated enhanced inflammation in the gastrocnemius of the *Mcp-1*-Tg mice as compared with WT mice, as illustrated by infiltration of macrophages between the muscle fibres (Fig. 1d). In addition, mRNA levels of *Il1b* and the macrophage markers *F4/80* and *Cd68* were significantly increased in the gastrocnemius of the *Mcp-1*-Tg mice (Fig. 1e). In contrast, expression of the same inflammatory markers in liver (Fig. 1f) and WAT (Fig. 1g) did not reveal any differences between WT and *Mcp-1*-Tg mice.

**Fig. 2:**
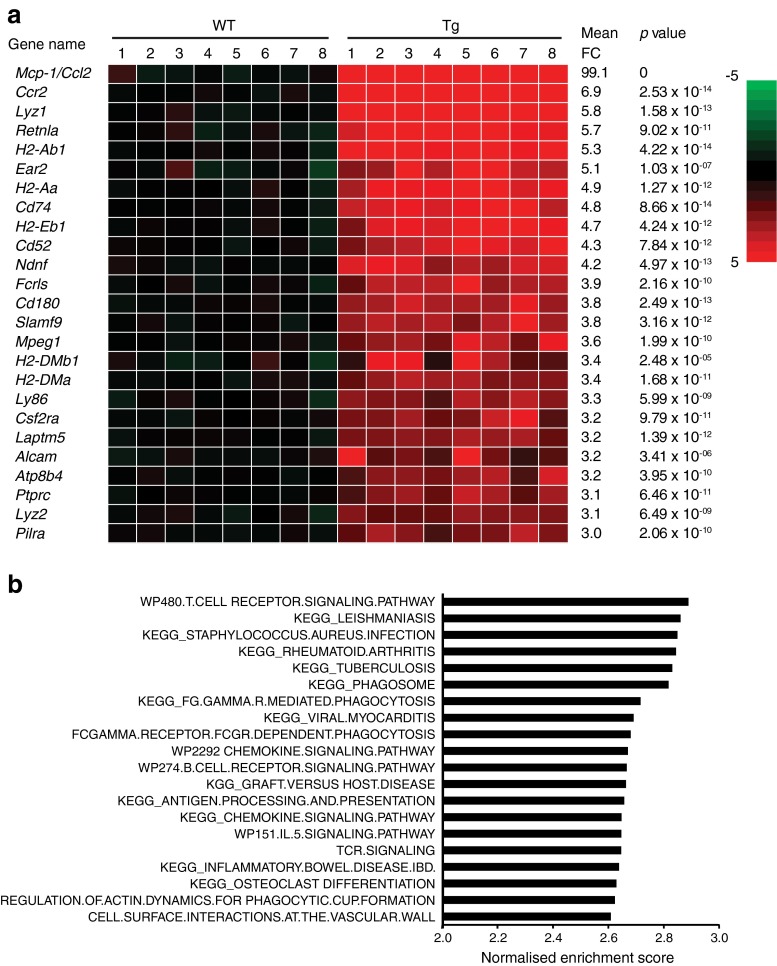
MCP-1 overexpression induces an inflammatory gene expression profile. (**a**) Gene expression changes in gastrocnemius muscle, illustrated by a heatmap of the 25 most highly induced genes in *Mcp-1*-Tg mice compared with WT mice. FC, fold change. (**b**) Gene sets positively enriched in the gastrocnemius of *Mcp-1*-Tg mice ranked according to normalised enrichment score (GSEA). The enrichment score reflects the degree to which a gene set is over-represented at the top or bottom of a ranked list of genes. Normalisation accounts for differences in gene set size and in correlations between gene sets and the expression dataset

Next, we performed microarray analysis on the gastrocnemius of eight WT mice and eight *Mcp-1*-Tg mice to study the overall effect of MCP-1 overexpression on gene expression in skeletal muscle. Overexpression of MCP-1 caused a clear inflammatory gene expression signature (Fig. 2a). The 25 most highly induced genes were all involved in inflammation and/or immunity, including the genes encoding lysozyme *(Lyz1)*, the immune cells surface markers *Cd52* and *Cd180*, and the secreted protein *Retnla*. Interestingly, the most highly induced gene was *Mcp-1* (99-fold), followed by its receptor *Ccr2*. Gene set enrichment analysis (GSEA) corroborated the pronounced induction of inflammation and immune-related pathways. Specifically, the top 20 of most highly enriched pathways are all linked to inflammation, including chemokine signalling, T cell receptor signalling, phagocytosis and inflammatory disease-related pathways (Fig. 2b). The complete list of significantly regulated genes (*q* value < 0.05) is presented in ESM Table 2. Taken together, these data indicate that MCP-1 overexpression promotes inflammation in skeletal muscle.

### No metabolic phenotype can be observed in mice overexpressing MCP-1 in skeletal muscle when fed a normal chow diet

**Fig. 3:**
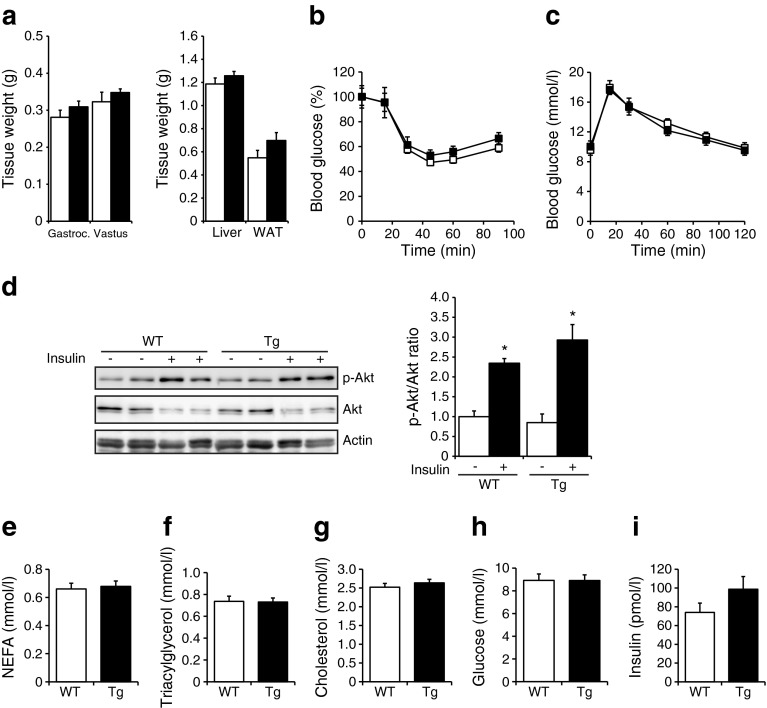
No metabolic phenotype is observed in mice overexpressing *Mcp-1* in skeletal muscle under normal chow diet. (**a**) Tissue weights of musculus gastrocnemius (Gastroc.), musculus vastus, liver and WAT; *n* = 8 (WT mice) or *n* = 11 (*Mcp-1*-Tg mice). (**b**, **c**) Blood glucose level during insulin tolerance test (**b**) and glucose tolerance test (**c**); *n* = 8 (WT and *Mcp-1*-Tg mice). (**d**) Representative western blot showing phosphorylation levels of Akt in gastrocnemius of WT and *Mcp-1*-Tg mice upon insulin injection. Bar graph shows quantification of p-Akt/Akt ratio performed in four mice per treatment group and normalised to WT mice without insulin. (**e**–**i**) Plasma concentration of NEFA (**e**), triacylglycerol (**f**), cholesterol (**g**), glucose (**h**) and insulin (**i**); *n* = 8 (WT mice) or *n* = 11 (*Mcp-1*-Tg mice). White bars and squares, WT mice; black bars and squares, *Mcp-1*-Tg mice. Data are means ± SEM; *p < 0.05 according to Student’s *t* test for insulin-treated mice vs control-treated mice (**d**)

To test whether skeletal muscle-specific overexpression of MCP-1 and associated inflammation may also influence glucose homeostasis and insulin sensitivity, *Mcp-1*-Tg mice and their WT littermates were subjected to detailed metabolic characterisation. *Mcp-1*-Tg and WT mice had similar weights of musculus gastrocnemius, musculus vastus, liver and WAT (Fig. 3a). Intriguingly, the results of an intraperitoneal insulin tolerance test (Fig. 3b) and intraperitoneal glucose tolerance test (Fig. 3c) did not differ between *Mcp-1*-Tg and WT mice. In addition, we analysed the effect of muscle-specific MCP-1 overexpression on the sensitivity of muscle to i.p. injection of insulin, using phosphorylation of Akt as a readout. Insulin injection markedly stimulated Akt phosphorylation in the gastrocnemius muscle (Fig. 3d). However, no significant difference in phospho-Akt could be observed between WT and *Mcp-1*-Tg mice, indicating that MCP-1 overexpression does not alter insulin-dependent signalling in skeletal muscle. Finally, the *Mcp-1*-Tg mice did not show any changes in plasma concentrations of NEFA, triacylglycerol, cholesterol, glucose or insulin when compared with WT mice (Fig. 3e–i). Taken together, our results indicate a lack of an effect of muscle-specific MCP-1 overexpression on insulin sensitivity and glucose homeostasis in mice fed a normal chow diet.

### HFD feeding does not result in metabolic alterations in muscle-specific *Mcp-1*-Tg mice

**Fig. 4:**
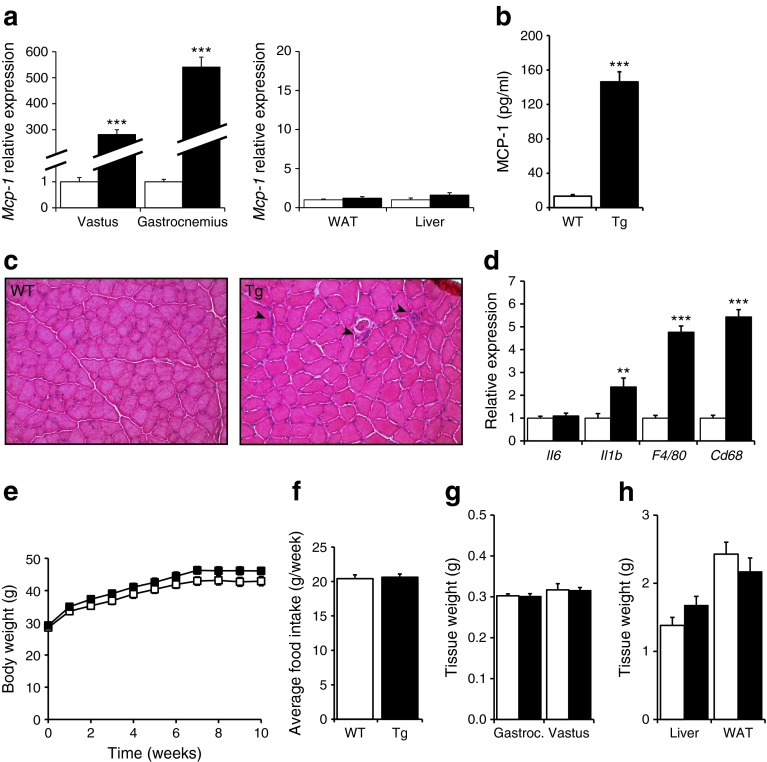
Characterisation of muscle-specific *Mcp-*1-Tg mice fed an HFD. (**a**) Relative gene expression of *Mcp-1* measured by qPCR. (**b**) Blood concentration of MCP-1. (**c**) H&E staining of representative sections of gastrocnemius of WT and *Mcp-1*-Tg mice (magnification ×200); Arrowheads indicate macrophage infiltration. (**d**) Relative gene expression of *Il6*, *Il1b*, *F4/80* and *Cd68* measured by qPCR. (**e**): Body weight changes in the WT and *Mcp-1*-Tg mice. (**f**) Average food intake from week 2 to week 8. (**g**) Tissue weights of musculus gastrocnemius (Gastroc.), musculus vastus, liver and WAT. White bars and squares, WT mice; black bars and squares, *Mcp-1*-Tg mice. Data are means ± SEM; *n* = 11 (WT mice) or *n* = 12 (*Mcp-1*-Tg mice); **p < 0.01 and ***p < 0.001 according to Student’s *t* test for WT vs *Mcp-1*-Tg mice

**Fig. 5:**
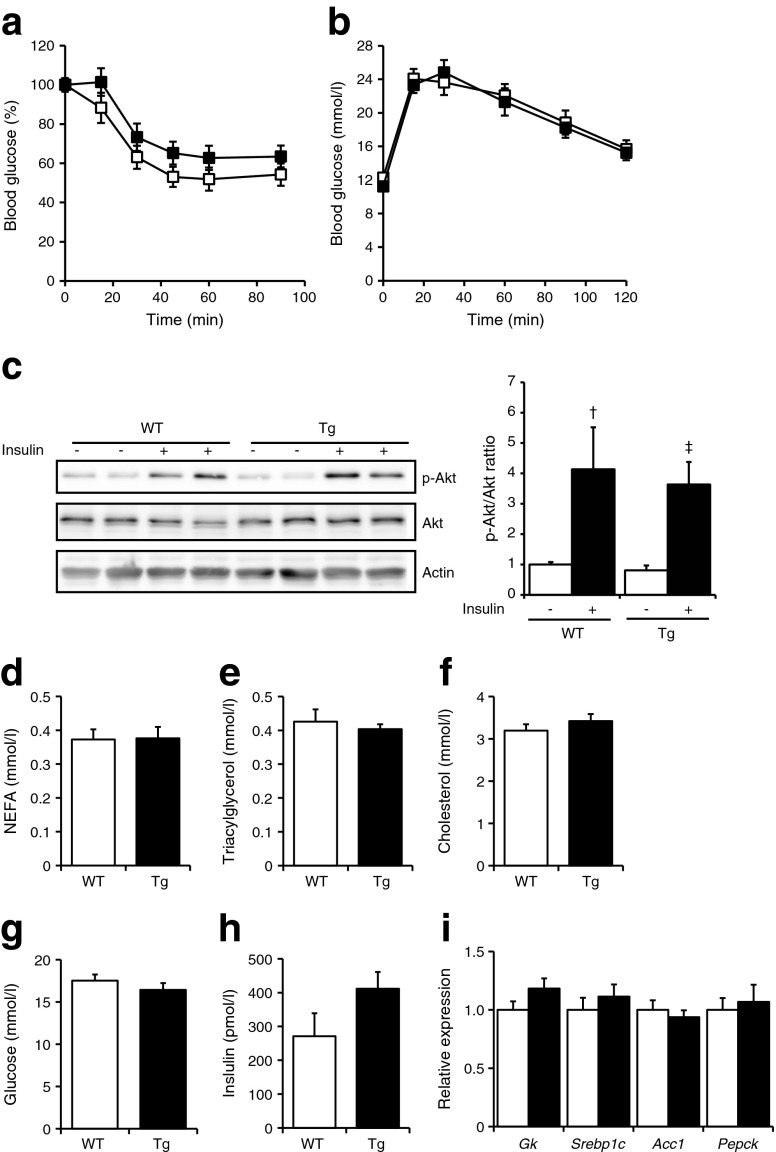
HFD feeding does not result in metabolic alterations in muscle-specific *Mcp-1*-Tg mice. (**a**, **b**) Plasma glucose level during insulin tolerance test (**a**) and glucose tolerance test (**b**); *n* = 11 (WT mice) or *n* = 12 (*Mcp-1*-Tg mice). (**c**) Representative western blot showing phosphorylation levels of Akt in gastrocnemius of WT and *Mcp-1*-Tg mice upon injection of insulin. Bar graph shows quantification of p-Akt/Akt ratio performed on two mice per treatment group and normalised to WT mice without insulin. (**d**–**h**) Plasma concentration of NEFA (**d**), triacylglycerol (**e**), cholesterol (**f**), glucose (**g**) and insulin (**h**); *n* = 7 (WT mice) or *n* = 8 (*Mcp-1*-Tg mice). (**i**) Relative gene expression of *Gk*, *Srebp1c*, *Acc1* and *Pepck* in liver measured by qPCR; *n* = 12 (for WT and *Mcp-1*-Tg mice). White bars and squares, WT mice; black bars and squares, *Mcp-1*-Tg mice. Data are means ± SEM; ^†^ *p* = 0.08, ^‡^ *p* = 0.03 according to Student’s *t* test for insulin-treated mice vs control-treated mice (**c**)

One possible explanation for the lack of effect of MCP-1 overexpression on variables associated with insulin sensitivity is related to the fact that the mice were fed normal chow and were therefore lean. To determine the impact of muscle-specific overexpression of MCP-1 on glucose homeostasis and insulin sensitivity in the context of obesity and insulin resistance, muscle-specific *Mcp-1*-Tg and WT littermates were rendered obese and insulin resistant by chronic high-fat feeding. The muscle-specific overexpression of *Mcp-1* in the *Mcp-1*-Tg mice was maintained after HFD (Fig. 4a), accompanied by a 10.9-fold increase in plasma MCP-1 levels (Fig. 4b). H&E staining showed more pronounced macrophage infiltration in the gastrocnemius of the *Mcp-1*-Tg mice compared with WT mice (Fig. 4c). In addition, mRNA levels of *IL1b* and the macrophage markers *F4/80* and *Cd68* were significantly increased in the gastrocnemius of the *Mcp-1*-Tg mice (Fig. 4d). In contrast, expression of inflammatory markers remained unchanged in liver and adipose tissue of the *Mcp-1*-Tg mice, with the exception of macrophage marker *F4/80* and alternatively-activated macrophage marker *Cd206*, which were both significantly reduced in liver of *Mcp-1*-Tg mice (ESM Fig. 1). Whole-body weight (Fig. 4e) and food intake (Fig. 4f) did not differ between *Mcp-1*-Tg mice and WT mice on HFD, and there was no difference in weights of the musculus gastrocnemius, musculus vastus, liver and WAT (Fig. 4g). An intraperitoneal insulin tolerance test and glucose tolerance test revealed decreased glucose tolerance in mice fed HFD compared with chow. However, no differences in insulin and glucose tolerance were detected when comparing diet-induced obese *Mcp-1*-Tg mice with WT mice (Fig. 5a, b). As observed in chow-fed mice, insulin-induced Akt phosphorylation was similar in the gastrocnemius of HFD-fed *Mcp-1*-Tg mice compared with HFD-fed WT mice (Fig. 5c). In addition, none of the plasma metabolites measured was significantly changed in the *Mcp-1*-Tg mice compared with WT mice. (Fig. 5d–h). Finally, hepatic expression of insulin-sensitive genes *Gk*, *Srebp1c* (also known as *Srebf1*), *Acc1* (*Acaca*), and *Pepck* (*Pck1*) was unaffected by muscle-specific MCP-1 overexpression (Fig. 5i). Taken together, muscle-specific overexpression of MCP-1 did not affect insulin sensitivity and glucose homeostasis in obese insulin-resistant mice.

## Discussion

The aim of the present study was to assess to what extent elevation of MCP-1 production in muscle and elevated MCP-1 levels in plasma interfere with insulin signalling in skeletal muscle and promote insulin resistance. To this end, an *Mck-Mcp-1*-Tg mouse model that specifically overexpresses MCP-1 in skeletal muscle was used. We show that muscle-specific overexpression of MCP-1 promotes inflammation in skeletal muscle but does not influence insulin signalling in skeletal muscle and has no effect on whole-body insulin sensitivity and glucose tolerance in either lean or obese mice. These findings therefore indicate that muscle inflammation by itself is not sufficient to evoke whole-body insulin resistance.

Studies to date have not been consistent in establishing a causal relation between muscle-specific stimulation or repression of inflammatory molecules and alterations in insulin sensitivity. On the one hand, it has been shown that over-production of constitutively activated c-Jun N-terminal kinase in skeletal muscle impairs insulin signalling in mice by modulating serine and threonine phosphorylation of Akt and IRS-1 [28]. On the other hand, muscle-specific ablation of IκB kinase 2, resulting in reduced activation of the transcription factor nuclear factor-κB (NF-κB), had no effect on obesity-induced insulin resistance and on insulin signalling in muscle [29]. Similarly, muscle-specific ablation of signal transducer and activator of transcription 3 (STAT3), another transcription factor activated by proinflammatory cytokines, showed no effect on obesity-induced insulin resistance, as assessed by glucose tolerance test, clamp studies and measurement of 2-deoxyglucose uptake [30]. Our study indicates that local activation of inflammation in muscle via MCP-1 overexpression does not affect insulin sensitivity and insulin action. Along the same lines, overexpression of NF-κB subunit p65 and IκBKβ in single muscles of rats using in vivo electrotransfer had no effect on glucose disposal in muscle under hyperinsulinaemic–euglycaemic clamp conditions, suggesting that activation of the IκBKβ–NF-κB pathway in muscle does not seem to be an important local mediator of insulin resistance [31].

Presently, it is difficult to merge the diverse findings into a coherent and consistent picture describing the role and impact of inflammatory signalling in muscle on local insulin resistance. A provocative conclusion emerging from our study and other ‘negative’ studies is that obesity-induced insulin resistance in muscle, the primary organ accounting for insulin-mediated glucose disposal, is not dependent on inflammatory signalling in muscle itself; this in turn can be extrapolated to suggest that development of insulin resistance in muscle during obesity does not require inflammatory mediators that affect skeletal muscle. In future studies, more attention should be accorded to clarification of the role of inflammatory signalling from adipose tissue to muscle in the development of insulin resistance.

In contrast to our findings, Patsouris et al observed modest whole-body and muscle insulin resistance and mildly elevated plasma glucose levels in *Mck–Mcp-1*-Tg mice [25]. One of the potential reasons for the discrepancy between our data and those of Patsouris is the composition of the background diet, as our chow diet and HFD are different from the diets used by Patsouris et al. Another important difference between our study and the study by Patsouris and colleagues is that we measured plasma metabolic variables (i.e. insulin, glucose, triacylglycerol) in 6 h-fasted mice, whereas Patsouris assessed the same variables in fed mice. Unfortunately, additional relevant experimental details (number of mice used, sex of the mice, time of euthanasia) are not mentioned in the Patsouris paper, which would have allowed for a more detailed comparison between the two studies [25].

In contrast to the present study in mice with muscle-specific overexpression of MCP1, studies performed in mice with adipose tissue-specific overexpression of MCP-1 or expression of a dominant-negative mutant of MCP-1 show that adipose tissue-derived MCP-1 reduces glucose tolerance and insulin sensitivity and blunts insulin-mediated signalling in skeletal muscle [11, 12]. Also, in vitro treatment of human skeletal muscle cells or the C2C12 muscle cell line with recombinant MCP-1 protein results in decreased Akt phosphorylation upon insulin stimulation [10, 11]. This raises the question why MCP-1 secreted from adipose tissue is able to alter whole-body and muscle insulin sensitivity whereas MCP-1 secreted from the muscle is not, especially since the levels of plasma MCP-1 were comparable in both transgenic mouse models [11, 12]. One explanation may be that circulating MCP-1 only plays a minor role in regulating whole-body insulin sensitivity and that local MCP-1 concentrations predominantly determine the metabolic outcome: raising MCP-1 levels in muscle only results in local inflammation, while increased MCP-1 expression in adipose tissue starts a cascade of events including altered adipose-resident immune cell profiles and altered output of adipokines and metabolites, ultimately resulting in impaired whole-body insulin sensitivity. It should be noted, however, that acute or chronic elevation of circulating MCP-1 by injection results in insulin resistance [32]. Accordingly, a possible alternative explanation is that circulating MCP-1 is able to affect insulin sensitivity but that the form secreted by adipose tissue is different from the form secreted by muscle tissue. It is known that MCP-1 exists in an unglycosylated (8–12 kDa) and in a glycosylated form (17–30 kDa) [33–35] and it has been suggested that the glycosylated form can bind to cell membranes, increasing the local MCP-1 expression, while unglycosylated MCP-1 is more easily diffusible and able to form a potent chemotactic gradient [36]. The MCP-1 protein detected by western blotting in this study has a molecular mass of approximately 18 kDa, which suggests that the MCP-1 expressed in skeletal muscle is glycosylated. Furthermore, in vitro treatment of muscle cells with MCP-1 and acute or chronic elevation of MCP-1 by injection in vivo, which both resulted in insulin resistance, were all performed with unglycosylated recombinant MCP-1 protein produced in *E. coli* [10, 11, 32]. More research is therefore needed to investigate the glycosylation status of MCP-1 originating from different tissues. Moreover, it would be interesting to determine whether MCP-1 glycosylation status influences insulin signalling in insulin-sensitive tissues.

By acting as a myokine and as an inflammatory mediator, MCP-1 displays characteristics similar to those of IL-6, the most studied and discussed myokine (reviewed in [22]). It has been suggested that IL-6 produced by skeletal muscle has a stimulatory influence on insulin sensitivity in skeletal muscle, whereas opposite effects have been shown for elevated IL-6 levels in adipose tissue and liver [23, 37–39]. Accordingly, the effect of both MCP-1 and IL-6 on insulin sensitivity seems to be very tissue-specific and may also depend on whether they are induced acutely or chronically [22]. Examining the parallels between MCP-1 and IL-6 may help unravel the underlying mechanism accounting for the tissue-specific actions of these two dual-faced regulators of glucose homeostasis.

A limitation of our study is that we only assessed muscle-specific insulin signalling at the level of Akt phosphorylation and did not examine other insulin-sensitive targets or perform clamp studies. Another limitation is that we cannot rule out that (obesity-induced) overexpression of MCP-1 specifically during adulthood has a different effect on insulin action than permanent MCP-1 overexpression in muscle starting during embryonic development. Future studies using inducible *Mcp*-1-Tg mice are necessary to clarify this issue.

In conclusion, elevation of MCP-1 production in skeletal muscle and concomitant elevation of plasma MCP-1 lead to enhanced inflammation in skeletal muscle but have no effect on insulin resistance and glucose tolerance in lean and obese mice and do not affect insulin-mediated signalling in skeletal muscle.

## Electronic supplementary material

ESM Methods (PDF 87 kb)

ESM Fig. 1 (PDF 62 kb)

ESM Table 1 (PDF 81 kb)

ESM Table 2 (PDF 186 kb)

## Abbreviations

ER: Endoplasmic reticulum
GSEA: Gene set enrichment analysis
H&E: Hematoxylin & eosin
HFD: High-fat diet
MCK: Muscle creatine kinase
MCP-1: Monocyte chemoattractant protein 1
NF-κB: Nuclear factor-κB
qPCR: Quantitative real-time PCR
Tg: Transgenic
WAT: White adipose tissue
WT: Wild-type
ZT: Zeitgeber time
