# Supplementary material for: Muscle-specific inflammation induced by MCP-1 overexpression does not affect whole-body insulin sensitivity in mice
Source: Diabetologia. 2015 Dec 12;59:624–33. doi: 10.1007/s00125-015-3822-2 (PMC4742493; doi:10.1007/s00125-015-3822-2)
Supplement: Supplementary file 1 — (PDF 87 kb) [file 125_2015_3822_MOESM1_ESM.pdf]

ESM Methods

Affymetrix microarray

Microarray analysis was performed on RNA of the musculus gastrocnemius. The RNA of 8 WT mice and 8 Tg mice was extracted using TRIzol reagent (Invitrogen, Carlsbad, CA, USA) and a Tissuelyser II (Qiagen, Germantown, MD, USA), purified using the RNeasy Micro Kit (Qiagen) and the integrity were verified with the RNA 6000 Nano assay on the Agilent 2100 Bioanalyzer (Agilent Technologies, Amsterdam, the Netherlands). Hybridization, washing, and scanning of the Affymetrix GeneChip Mouse Gene 1.1 ST Array were performed on Affymetrix GeneTitan. Scans of the Affymetrix arrays were processed using packages from the Bioconductor project [1]. Arrays were normalized and raw signal intensities were obtained using the Robust Multiarray Average method [2, 3]. Probe sets were defined according to Dai et al. [4]. In this method probes are assigned to Entrez IDs as a unique gene identifier. The P values were calculated using an Intensity-Based Moderated T-statistic (IBMT) [5]. The microarray data were submitted to the Gene Expression Omnibus (accession number pending). Gene set enrichment analysis (GSEA) was used to find enriched gene sets in the induced or suppressed genes [6]. Genes were ranked based on the IBMT-statistic and subsequently analyzed for over- or underrepresentation in predefined gene sets derived from Gene Ontology, KEGG, National Cancer Institute, PFAM, Biocarta, Reactome and WikiPathways pathway databases. Only gene sets consisting of more than 15 and fewer than 500 genes were taken into account. Statistical significance of GSEA results was determined using 1,000 permutations.

References

1. Gentleman RC, Carey VJ, Bates DM et al (2004) Bioconductor: Open software development for computational biology and bioinformatics. Genome Biol 5:R80
2. Bolstad BM, Irizarry RA, Astrand M and Speed TP (2003) A comparison of normalization methods for high density oligonucleotide array data based on variance and bias. Bioinformatics 19:185-193
3. Irizarry RA, Bolstad BM, Collin F, Cope LM, Hobbs B and Speed TP (2003) Summaries of affymetrix genechip probe level data. Nucleic Acids Res 31:e15
4. Dai M, Wang P, Boyd AD et al (2005) Evolving gene/transcript definitions significantly alter the interpretation of genechip data. Nucleic Acids Res 33:e175
5. Sartor MA, Tomlinson CR, Wesselkamper SC, Sivaganesan S, Leikauf GD and Medvedovic M (2006) Intensity-based hierarchical bayes method improves testing for differentially expressed genes in microarray experiments. BMC Bioinformatics 7:538
6. Subramanian A, Tamayo P, Mootha VK et al (2005) Gene set enrichment analysis: A knowledge-based approach for interpreting genome-wide expression profiles. Proc Natl Acad Sci U S A 102:15545-15550
