# Supplementary material for: Muscle-specific inflammation induced by MCP-1 overexpression does not affect whole-body insulin sensitivity in mice
Source: Diabetologia. 2015 Dec 12;59:624–33. doi: 10.1007/s00125-015-3822-2 (PMC4742493; doi:10.1007/s00125-015-3822-2)
Supplement: Supplementary file 2 — (PDF 62 kb) [file 125_2015_3822_MOESM2_ESM.pdf]

ESM Fig. 1

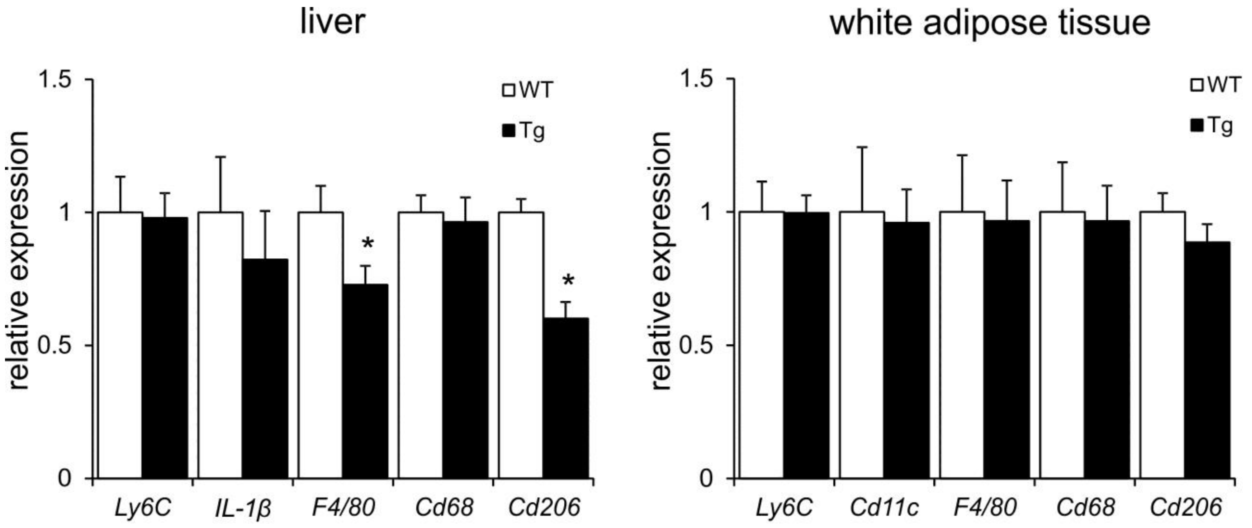

Supplementary figure 1: Relative gene expression of selected genes in liver and white adipose tissue measured by RT-PCR; n = 12 [WT], 12 [MCP-1-Tg]. Data are means ± SEM; * = $p < 0.05$;
