# Supplementary material for: Muscle-specific inflammation induced by MCP-1 overexpression does not affect whole-body insulin sensitivity in mice
Source: Diabetologia. 2015 Dec 12;59:624–33. doi: 10.1007/s00125-015-3822-2 (PMC4742493; doi:10.1007/s00125-015-3822-2)
Supplement: Supplementary file 3 — (PDF 81 kb) [file 125_2015_3822_MOESM3_ESM.pdf]

ESM Table 1

List of real-time PCR primers

Gene	Forward	Reverse
**m36B4**	AGCGCGTCCTGGCATTGTCTGTGG	GGGCAGCAGTGGTGGCAGCAGC
**mCd68**	CCAATTCAGGGTGGAAGAAA	CTCGGGCTCTGATGTAGGTC
**mF4/80**	CTTTGGCTATGGGCTTCCAGTC	GCAAGGAGGACAGAGTTTATCGTG
**mIl-1β**	CAGGCAGGCAGTATCACTCA	AGGTGCTCATGTCCTCATCC
**mIl-6**	CTTCCATCCAGTTGCCTTCTTG	AATTAAGCCTCCGACTTGTGAAG
