# Supplementary material for: Muscle-specific inflammation induced by MCP-1 overexpression does not affect whole-body insulin sensitivity in mice
Source: Diabetologia. 2015 Dec 12;59:624–33. doi: 10.1007/s00125-015-3822-2 (PMC4742493; doi:10.1007/s00125-015-3822-2)
Supplement: Supplementary file 4 — (PDF 186 kb) [file 125_2015_3822_MOESM4_ESM.pdf]

ESM Table 2

Genes significantly altered between gastrocnemius of WT and MCP-1-Tg mice (q value<0.05)

Entrez ID	Gene name	Fold change	q value	Description
20296	Ccl2	99.11	0.00E+00	chemokine (C-C motif) ligand 2
12772	Ccr2	6.90	2.09E-10	chemokine (C-C motif) receptor 2
17110	Lyz1	5.76	5.22E-10	lysozyme 1
57262	Retnla	5.73	6.48E-08	resistin like alpha
14961	H2-Ab1	5.34	2.33E-10	histocompatibility 2, class II antigen A, beta 1
13587	Ear2	5.08	2.10E-05	eosinophil-associated, ribonuclease A family, member 2
14960	H2-Aa	4.88	2.55E-09	histocompatibility 2, class II antigen A, alpha
16149	Cd74	4.79	3.58E-10	CD74 antigen (invariant polypeptide ofMHC, class II antigen-associated)
14969	H2-Eb1	4.74	5.85E-09	histocompatibility 2, class II antigen E beta
23833	Cd52	4.31	8.65E-09	CD52 antigen
68169	Ndnf	4.17	1.17E-09	neuron-derived neurotrophic factor
80891	Fcrls	3.87	1.23E-07	Fc receptor-like S, scavenger receptor
17079	Cd180	3.81	6.87E-10	CD180 antigen
98365	Slamf9	3.80	5.23E-09	SLAM family member 9
17476	Mpeg1	3.58	1.22E-07	macrophage expressed gene 1
14999	H2-DMb1	3.43	2.14E-03	histocompatibility 2, class II, locus Mb1
14998	H2-DMa	3.36	1.64E-08	histocompatibility 2, class II, locus DMa
17084	Ly86	3.32	1.80E-06	lymphocyte antigen 86
12982	Csf2ra	3.24	6.48E-08	colony stimulating factor 2 receptor, alpha, low-affinity (granulocyte-macrophage)
16792	Laptm5	3.22	2.55E-09	lysosomal-associated protein transmembrane 5
11658	Alcam	3.21	4.03E-04	activated leukocyte cell adhesion molecule
241633	Atp8b4	3.15	1.98E-07	ATPase, class I, type 8B, member 4
19264	Ptprc	3.12	4.85E-08	protein tyrosine phosphatase, receptor type, C
17105	Lyz2	3.07	1.82E-06	lysozyme 2
231805	Pilra	3.02	1.22E-07	paired immunoglobin-like type 2 receptor alpha
12523	Cd84	2.99	1.82E-06	CD84 antigen
213391	Rassf4	2.98	4.85E-08	Ras association (RalGDS/AF-6) domain family member 4
13723	Emb	2.98	5.93E-06	embigin
12508	Cd53	2.91	2.77E-07	CD53 antigen
227929	Cytip	2.88	1.52E-06	cytohesin 1 interacting protein
104759	Pld4	2.88	9.84E-08	phospholipase D family, member 4
216864	Mgl2	2.85	3.28E-08	macrophage galactose N-acetyl-galactosamine specific lectin 2
225825	Cd226	2.84	1.29E-06	CD226 antigen
213002	Ifitm6	2.81	8.04E-06	interferon induced transmembrane protein 6
17381	Mmp12	2.75	2.52E-06	matrix metallopeptidase 12
20345	Selplg	2.75	2.37E-07	selectin, platelet (p-selectin) ligand
217305	Cd300ld	2.73	1.92E-05	CD300 molecule-like family member d
72318	Cyth4	2.73	5.85E-09	cytohesin 4
16414	Itgb2	2.72	4.85E-08	integrin beta 2
18726	Lilra6	2.70	6.53E-04	leukocyte immunoglobulin-like receptor, subfamily A (with TM domain), member 6
94176	Dock2	2.70	1.25E-07	dedicator of cyto-kinesis 2
216984	Evi2b	2.68	1.52E-06	ecotropic viral integration site 2b
16409	Itgam	2.66	6.60E-07	integrin alpha M
100038909	Gm14548	2.64	1.01E-03	predicted gene 14548
20375	Spi1	2.64	8.65E-09	spleen focus forming virus (SFFV) proviral integration oncogene
12721	Coro1a	2.59	2.37E-07	coronin, actin binding protein 1A
414084	Tnip3	2.58	1.42E-07	TNFAIP3 interacting protein 3
14728	Lilrb4	2.58	1.29E-06	leukocyte immunoglobulin-like receptor, subfamily B, member 4
19260	Ptpn22	2.55	1.25E-07	protein tyrosine phosphatase, non-receptor type 22 (lymphoid)
14727	Gp49a	2.55	5.40E-05	glycoprotein 49 A
319446	Dpep2	2.54	1.70E-06	dipeptidase 2
627984	Nlrp1c-ps	2.54	1.13E-05	NLR family, pyrin domain containing 1C, pseudogene
20308	Ccl9	2.52	8.11E-06	chemokine (C-C motif) ligand 9
13058	Cybb	2.50	1.44E-06	cytochrome b-245, beta polypeptide
394432	Ugt1a7c	2.49	2.60E-04	UDP glucuronosyltransferase 1 family, polypeptide A7C
11690	Alox5ap	2.48	2.04E-06	arachidonate 5-lipoxygenase activating protein
20305	Ccl6	2.48	7.78E-05	chemokine (C-C motif) ligand 6
13040	Ctss	2.46	5.68E-06	cathepsin S
18636	Cfp	2.46	1.80E-06	complement factor properdin
74039	Nfam1	2.45	5.98E-07	Nfat activating molecule with ITAM motif 1

11501	Adam8	2.43	6.23E-09	a disintegrin and metallopeptidase domain 8
69810	Clec4b1	2.43	3.50E-03	C-type lectin domain family 4, member b1
232413	Clec12a	2.43	1.00E-05	C-type lectin domain family 12, member a
66815	Ccdc109b	2.40	8.55E-07	coiled-coil domain containing 109B
230787	Themis2	2.37	1.52E-06	thymocyte selection associated family member 2
18830	Pltp	2.37	1.15E-08	phospholipid transfer protein
14127	Fcer1g	2.35	6.48E-08	Fc receptor, IgE, high affinity I, gamma polypeptide
16822	Lcp2	2.33	4.33E-07	lymphocyte cytosolic protein 2
56644	Clec7a	2.31	3.86E-05	C-type lectin domain family 7, member a
13449	Dok2	2.28	1.71E-06	docking protein 2
21391	Tbxas1	2.27	3.45E-07	thromboxane A synthase 1, platelet
107684	Coro2a	2.24	2.12E-05	coronin, actin binding protein 2A
12265	Ciita	2.24	1.37E-06	class II transactivator
15000	H2-DMb2	2.23	6.28E-04	histocompatibility 2, class II, locus Mb2
19204	Ptafr	2.21	3.73E-06	platelet-activating factor receptor
18826	Lcp1	2.20	4.02E-05	lymphocyte cytosolic protein 1
19277	Ptpro	2.18	1.01E-02	protein tyrosine phosphatase, receptor type, O
72042	Cotl1	2.17	2.71E-08	coactosin-like 1 (Dictyostelium)
100038363	RIKEN	2.16	1.44E-03	RIKEN cDNA F630028O10 gene
12514	Cd68	2.15	4.84E-05	CD68 antigen
75345	Slamf7	2.11	1.03E-05	SLAM family member 7
105855	Nckap1l	2.11	2.18E-07	NCK associated protein 1 like
18301	Fxyd5	2.10	1.37E-05	FXYD domain-containing ion transport regulator 5
212937	Tifab	2.10	6.98E-06	TRAF-interacting protein with forkhead-associated domain, family member B
12481	Cd2	2.09	1.82E-06	CD2 antigen
18796	Plcb2	2.08	7.80E-05	phospholipase C, beta 2
16854	Lgals3	2.06	7.78E-05	lectin, galactose binding, soluble 3
12260	C1qb	2.05	8.60E-06	complement component 1, q subcomponent, beta polypeptide
22177	Tyrobp	2.05	6.02E-04	TYRO protein tyrosine kinase binding protein
73149	Clec4a3	2.04	1.03E-04	C-type lectin domain family 4, member a3
12493	Cd37	2.02	1.00E-05	CD37 antigen
19354	Rac2	2.01	1.54E-03	RAS-related C3 botulinum substrate 2
503550	Klri1	1.95	1.66E-04	killer cell lectin-like receptor family I member 1
56792	Stap1	1.95	1.00E-05	signal transducing adaptor family member 1
20491	Sla	1.94	2.10E-05	src-like adaptor
26904	Sh2d1b1	1.94	4.48E-03	SH2 domain protein 1B1
226652	Arhgap30	1.92	2.69E-05	Rho GTPase activating protein 30
76088	Dock8	1.91	1.29E-06	dedicator of cytokinesis 8
98496	Pid1	1.89	1.15E-06	phosphotyrosine interaction domain containing 1
12506	Cd48	1.89	2.82E-05	CD48 antigen
54445	Unc93b1	1.89	3.12E-05	unc-93 homolog B1 (C. elegans)
668218	Bin2	1.89	1.07E-03	bridging integrator 2
22778	Ikzf1	1.89	2.30E-05	IKAROS family zinc finger 1
20351	Sema4a	1.89	2.83E-05	Semaphorin 4A
17969	Ncf1	1.88	8.81E-05	neutrophil cytosolic factor 1
58861	Cysltr1	1.87	7.30E-04	cysteinyl leukotriene receptor 1
19267	Ptpre	1.87	5.72E-05	protein tyrosine phosphatase, receptor type, E
52614	Emr4	1.87	2.71E-03	EGF-like module containing, mucin-like, hormone receptor-like sequence 4
78416	Rnase6	1.87	2.30E-05	ribonuclease, RNase A family, 6
17345	Mki67	1.86	1.51E-03	antigen identified by monoclonal antibody Ki 67
26411	Map4k1	1.86	1.37E-05	mitogen-activated protein kinase kinase kinase kinase 1
56177	Olfm1	1.85	1.78E-02	olfactomedin 1
14421	B4galnt1	1.84	1.25E-03	beta-1,4-N-acetyl-galactosaminyl transferase 1
73723	Sh3bgrl3	1.84	2.83E-05	SH3 domain binding glutamic acid-rich protein-like 3
64381	Ms4a8a	1.83	1.50E-03	membrane-spanning 4-domains, subfamily A, member 8A
239217	Kctd12	1.82	5.93E-06	potassium channel tetramerisation domain containing 12
320139	Ptpn7	1.81	1.39E-03	protein tyrosine phosphatase, non-receptor type 7
14130	Fcgr2b	1.80	1.61E-04	Fc receptor, IgG, low affinity IIb
16364	Irf4	1.79	2.80E-02	interferon regulatory factor 4
18718	Pip4k2a	1.79	6.47E-05	phosphatidylinositol-5-phosphate 4-kinase, type II, alpha
80719	Igsf6	1.79	4.62E-04	immunoglobulin superfamily, member 6
17916	Myo1f	1.79	7.78E-05	myosin IF
545030	Wdfy4	1.79	2.10E-04	WD repeat and FYVE domain containing 4
14131	Fcgr3	1.79	3.37E-03	Fc receptor, IgG, low affinity III
81897	Tlr9	1.78	6.31E-05	toll-like receptor 9
65963	Tmem176b	1.78	2.35E-05	transmembrane protein 176B
14017	Evi2a	1.77	2.55E-04	ecotropic viral integration site 2a
11810	Apobec1	1.77	1.37E-05	apolipoprotein B mRNA editing enzyme, catalytic polypeptide 1
246177	Myo1g	1.77	1.16E-04	myosin IG
668139	Gm8995	1.77	1.38E-04	predicted gene 8995

20963	Syk	1.77	8.16E-04	spleen tyrosine kinase
71653	RIKEN	1.76	1.08E-04	RIKEN cDNA 4930506M07 gene
17082	Il1rl1	1.76	5.03E-04	interleukin 1 receptor-like 1
67111	Naaa	1.75	7.41E-07	N-acylethanolamine acid amidase
19261	Sirpa	1.75	6.28E-04	signal-regulatory protein alpha
20198	S100a4	1.73	9.85E-03	S100 calcium binding protein A4
170786	Cd209a	1.72	7.73E-03	CD209a antigen
210293	Dock10	1.71	4.24E-04	dedicator of cytokinesis 10
236312	Pyhin1	1.71	2.10E-04	pyrin and HIN domain family, member 1
269799	Clec4a1	1.71	4.91E-04	C-type lectin domain family 4, member a1
64214	Rgs18	1.71	1.00E-03	regulator of G-protein signaling 18
12229	Btk	1.70	3.35E-03	Bruton agammaglobulinemia tyrosine kinase
18173	Slc11a1	1.70	5.40E-05	solute carrier family 11 (proton-coupled divalent metal ion transporters), member 1
26888	Clec4a2	1.70	1.88E-03	C-type lectin domain family 4, member a2
69993	Chn2	1.70	2.22E-02	chimerin 2
234779	Plcg2	1.70	5.30E-04	phospholipase C, gamma 2
21844	Tiam1	1.70	2.60E-04	T cell lymphoma invasion and metastasis 1
57781	Cd200r1	1.70	1.54E-03	CD200 receptor 1
13732	Emp3	1.70	2.64E-05	epithelial membrane protein 3
22228	Ucp2	1.70	6.82E-04	uncoupling protein 2 (mitochondrial, proton carrier)
217304	Cd300lb	1.70	2.35E-04	CD300 antigen like family member B
16541	Napsa	1.69	6.67E-03	napsin A aspartic peptidase
12505	Cd44	1.69	1.52E-04	CD44 antigen
17444	Grap2	1.69	6.22E-05	GRB2-related adaptor protein 2
170744	Tlr8	1.68	8.45E-03	toll-like receptor 8
13733	Emr1	1.68	8.12E-03	EGF-like module containing, mucin-like, hormone receptor-like sequence 1
100504464	RIKEN	1.68	3.16E-04	RIKEN cDNA E230016K23 gene
72461	Prcp	1.67	6.22E-05	prolylcarboxypeptidase (angiotensinase C)
210029	Metrnl	1.67	1.59E-03	meteorin, glial cell differentiation regulator-like
14667	Gm2a	1.66	6.05E-06	GM2 ganglioside activator protein
14457	Gas7	1.66	6.22E-05	growth arrest specific 7
14191	Fgr	1.66	2.38E-05	Gardner-Rasheed feline sarcoma viral (Fgr) oncogene homolog
83490	Pik3ap1	1.66	1.96E-04	phosphoinositide-3-kinase adaptor protein 1
16331	Inpp5d	1.66	1.34E-04	inositol polyphosphate-5-phosphatase D
108101	Fermt3	1.65	1.17E-03	fermitin family homolog 3 (Drosophila)
16534	Kcnn4	1.65	2.07E-04	potassium intermediate/small conductance Ca-activ. channel, subfam N, member 4
66058	Tmem176a	1.65	7.78E-05	transmembrane protein 176A
15170	Ptpn6	1.65	2.56E-04	protein tyrosine phosphatase, non-receptor type 6
22350	Ezr	1.64	4.28E-05	ezrin
224829	Trerf1	1.63	5.29E-05	transcriptional regulating factor 1
224109	Nrros	1.62	4.92E-03	negative regulator of reactive oxygen species
74131	Sash3	1.61	3.11E-03	SAM and SH3 domain containing 3
19271	Ptprj	1.61	2.12E-05	protein tyrosine phosphatase, receptor type, J
12267	C3ar1	1.60	1.34E-03	complement component 3a receptor 1
238875	Gapt	1.59	7.38E-03	Grb2-binding adaptor, transmembrane
13057	Cyba	1.59	7.10E-03	cytochrome b-245, alpha polypeptide
70719	Hmha1	1.59	4.60E-03	histocompatibility (minor) HA-1
16154	Il10ra	1.58	4.03E-03	interleukin 10 receptor, alpha
75767	Rab11fip1	1.58	2.14E-03	RAB11 family interacting protein 1 (class I)
56470	Rgs19	1.58	1.58E-04	regulator of G-protein signaling 19
67742	Samsn1	1.57	3.52E-03	SAM domain, SH3 domain and nuclear localization signals, 1
320181	Fndc7	1.56	1.88E-05	fibronectin type III domain containing 7
382062	AB124611	1.56	2.51E-02	cDNA sequence AB124611
170741	Pilrb1	1.56	5.22E-03	paired immunoglobin-like type 2 receptor beta 1
72828	Ubash3b	1.55	6.61E-03	ubiquitin associated and SH3 domain containing, B
73690	Glipr1	1.55	2.50E-03	GLI pathogenesis-related 1 (glioma)
12978	Csf1r	1.54	1.80E-03	colony stimulating factor 1 receptor
239081	Tlr11	1.54	7.38E-03	toll-like receptor 11
14268	Fn1	1.54	1.15E-03	fibronectin 1
227541	Camk1d	1.54	8.16E-04	calcium/calmodulin-dependent protein kinase ID
74127	Krt80	1.54	1.24E-02	keratin 80
76117	Arhgap15	1.54	1.38E-04	Rho GTPase activating protein 15
72925	March1	1.53	8.45E-03	membrane-associated ring finger (C3HC4) 1
107373	Fam111a	1.53	1.65E-02	family with sequence similarity 111, member A
70536	Qpct	1.53	5.03E-04	glutaminyl-peptide cyclotransferase (glutaminyl cyclase)
211228	Lrrc25	1.53	7.46E-04	leucine rich repeat containing 25
64095	Gpr35	1.53	1.77E-04	G protein-coupled receptor 35
27056	Irf5	1.53	5.66E-03	interferon regulatory factor 5
225471	Ticam2	1.53	1.23E-02	toll-like receptor adaptor molecule 2
22324	Vav1	1.53	1.56E-02	vav 1 oncogene

64380	Ms4a4c	1.52	1.47E-02	membrane-spanning 4-domains, subfamily A, member 4C
69769	Tnfaip8l2	1.52	2.24E-04	tumor necrosis factor, alpha-induced protein 8-like 2
16970	Lrmp	1.52	6.02E-04	lymphoid-restricted membrane protein
16985	Lsp1	1.52	1.59E-03	lymphocyte specific 1
637515	Nlrp1b	1.52	1.69E-03	NLR family, pyrin domain containing 1B
320664	Cass4	1.51	1.05E-02	Cas scaffolding protein family member 4
19229	Ptk2b	1.51	2.02E-02	PTK2 protein tyrosine kinase 2 beta
64008	Aqp9	1.51	3.96E-04	aquaporin 9
27405	Abcg3	1.50	5.03E-03	ATP-binding cassette, sub-family G (WHITE), member 3
21810	Tgfbi	1.50	1.16E-04	transforming growth factor, beta induced
227659	Slc2a6	1.50	5.92E-03	solute carrier family 2 (facilitated glucose transporter), member 6
27984	Efhd2	1.50	4.66E-04	EF hand domain containing 2
14255	Flt3	1.49	1.45E-02	FMS-like tyrosine kinase 3
20970	Sdc3	1.49	2.92E-03	syndecan 3
11857	Arhgdib	1.49	4.91E-04	Rho, GDP dissociation inhibitor (GDI) beta
70785	Dennd1c	1.49	1.05E-02	DENN/MADD domain containing 1C
107321	Lpxn	1.48	2.83E-02	leupaxin
67865	Rgs10	1.48	2.89E-02	regulator of G-protein signalling 10
19735	Rgs2	1.48	4.85E-02	regulator of G-protein signaling 2
20556	Slfn2	1.48	8.03E-04	schlafen 2
320207	Pik3r5	1.48	1.54E-02	phosphoinositide-3-kinase, regulatory subunit 5, p101
215653	Rassf2	1.48	4.54E-02	Ras association (RalGDS/AF-6) domain family member 2
54135	Lsr	1.48	4.00E-03	lipolysis stimulated lipoprotein receptor
27007	Klrk1	1.48	5.03E-03	killer cell lectin-like receptor subfamily K, member 1
54519	Apbb1ip	1.47	1.91E-02	amyloid beta (A4) precursor protein-binding, family B, member 1 interacting protein
75766	Dcstamp	1.47	2.66E-02	dentrocyte expressed seven transmembrane protein
12475	Cd14	1.47	1.32E-02	CD14 antigen
56743	Lat2	1.47	5.66E-03	linker for activation of T cells family, member 2
17970	Ncf2	1.47	3.76E-02	neutrophil cytosolic factor 2
74748	Slamf8	1.47	1.13E-02	SLAM family member 8
26382	Fgd2	1.46	1.47E-02	FYVE, RhoGEF and PH domain containing 2
17952	Naip6	1.46	5.03E-03	NLR family, apoptosis inhibitory protein 6
68870	Ak8	1.46	6.61E-03	adenylate kinase 8
233046	Rasgrp4	1.45	4.58E-02	RAS guanyl releasing protein 4
544963	Iqgap2	1.45	1.44E-03	IQ motif containing GTPase activating protein 2
15163	Hcls1	1.45	9.46E-03	hematopoietic cell specific Lyn substrate 1
71607	Snx20	1.45	2.22E-02	sorting nexin 20
16633	Klra2	1.44	2.56E-04	killer cell lectin-like receptor, subfamily A, member 2
16411	Itgax	1.44	8.03E-04	integrin alpha X
102595	Plekho2	1.44	9.51E-04	pleckstrin homology domain containing, family O member 2
330177	Taok3	1.43	1.23E-03	TAO kinase 3
16194	Il6ra	1.43	1.29E-02	interleukin 6 receptor, alpha
101202	Hepacam2	1.43	1.25E-02	HEPACAM family member 2
21934	Tnfrsf11a	1.43	1.52E-02	tumor necrosis factor receptor superfamily, member 11a
11867	Arpc1b	1.43	7.38E-03	actin related protein 2/3 complex, subunit 1B
14269	Fnbp1	1.42	3.89E-02	formin binding protein 1
56193	Plek	1.42	6.61E-03	pleckstrin
23880	Fyb	1.42	1.28E-03	FYN binding protein
21803	Tgfb1	1.41	1.72E-02	transforming growth factor, beta 1
17095	Lyl1	1.41	2.29E-02	lymphoblastomic leukemia 1
29875	Iqgap1	1.41	4.71E-04	IQ motif containing GTPase activating protein 1
108686	Ccdc88a	1.41	4.74E-02	coiled coil domain containing 88A
57442	Kcne3	1.41	4.70E-03	potassium voltage-gated channel, Isk-related subfamily, gene 3
114644	Slc13a3	1.41	1.27E-02	solute carrier family 13 (sodium-dependent dicarboxylate transporter), member 3
242248	Bank1	1.41	1.26E-02	B cell scaffold protein with ankyrin repeats 1
380732	Milr1	1.41	4.54E-02	mast cell immunoglobulin like receptor 1
214854	Neurl3	1.41	1.16E-02	neuralized homolog 3 homolog (Drosophila)
19241	Tmsb4x	1.40	5.14E-04	thymosin, beta 4, X chromosome
383619	Aim2	1.40	4.13E-03	absent in melanoma 2
16535	Kcnq1	1.40	4.39E-02	potassium voltage-gated channel, subfamily Q, member 1
100182	Akna	1.40	1.94E-03	AT-hook transcription factor
72054	Cyp4f18	1.40	1.16E-02	cytochrome P450, family 4, subfamily f, polypeptide 18
18106	Cd244	1.39	4.31E-03	CD244 natural killer cell receptor 2B4
11513	Adcy7	1.39	6.06E-03	adenylate cyclase 7
233571	P2ry6	1.38	2.75E-02	pyrimidinergic receptor P2Y, G-protein coupled, 6
20868	Stk10	1.38	2.63E-02	serine/threonine kinase 10
54354	Rassf5	1.38	3.11E-02	Ras association (RalGDS/AF-6) domain family member 5
226421	RIKEN	1.38	5.08E-03	RIKEN cDNA 5430435G22 gene
12331	Cap1	1.38	7.96E-04	CAP, adenylate cyclase-associated protein 1 (yeast)
11630	Aim1	1.37	2.29E-02	absent in melanoma 1

233406	Prc1	1.37	1.32E-02	protein regulator of cytokinesis 1
56356	Gltp	1.37	8.46E-04	glycolipid transfer protein
12193	Zfp36l2	1.37	1.38E-02	zinc finger protein 36, C3H type-like 2
22376	Was	1.37	1.00E-03	Wiskott-Aldrich syndrome homolog (human)
23790	Coro1c	1.37	2.74E-04	coronin, actin binding protein 1C
57257	Vav3	1.36	4.48E-03	vav 3 oncogene
74735	Trim14	1.36	1.41E-02	tripartite motif-containing 14
13860	Eps8	1.36	5.27E-03	epidermal growth factor receptor pathway substrate 8
215632	Psd4	1.36	5.09E-02	pleckstrin and Sec7 domain containing 4
226101	Myof	1.35	2.20E-02	myoferlin
12798	Cnn2	1.34	6.53E-03	calponin 2
66494	Prelid1	1.34	3.43E-02	PRELI domain containing 1
107766	Haao	1.34	1.27E-02	3-hydroxyanthranilate 3,4-dioxygenase
223753	Cerk	1.34	4.07E-02	ceramide kinase
17972	Ncf4	1.34	4.67E-02	neutrophil cytosolic factor 4
56318	Acpp	1.34	4.78E-02	acid phosphatase, prostate
217203	Tmem106a	1.33	2.75E-02	transmembrane protein 106A
11461	Actb	1.32	7.13E-04	actin, beta
328660	Bex6	1.31	4.49E-02	brain expressed gene 6
12442	Ccnb2	1.31	3.59E-02	cyclin B2
12259	C1qa	1.31	4.93E-02	complement component 1, q subcomponent, alpha polypeptide
241197	Serpinb10	1.31	3.88E-02	serine (or cysteine) peptidase inhibitor, clade B (ovalbumin), member 10
71279	Slc29a3	1.29	8.22E-03	solute carrier family 29 (nucleoside transporters), member 3
16889	Lipa	1.28	3.89E-02	lysosomal acid lipase A
13710	Elf3	1.28	9.88E-03	E74-like factor 3
110835	Chrna5	1.28	3.10E-02	cholinergic receptor, nicotinic, alpha polypeptide 5
16643	Klrd1	1.27	2.97E-02	killer cell lectin-like receptor, subfamily D, member 1
330460	Tmem150b	1.27	4.44E-02	transmembrane protein 150B
223666	Arhgap39	1.27	4.00E-02	Rho GTPase activating protein 39
12489	Cd33	1.27	2.80E-02	CD33 antigen
244209	Cyp2r1	1.26	3.06E-02	cytochrome P450, family 2, subfamily r, polypeptide 1
238377	Gpr68	1.25	3.64E-02	G protein-coupled receptor 68
12306	Anxa2	1.24	2.38E-02	annexin A2
18438	P2rx4	1.23	2.75E-02	purinergic receptor P2X, ligand-gated ion channel 4
68598	Dnajc8	-1.23	2.29E-02	DnaJ (Hsp40) homolog, subfamily C, member 8
381816	RIKEN	-1.23	3.48E-02	RIKEN cDNA 4922502D21 gene
11529	Adh7	-1.24	4.66E-02	alcohol dehydrogenase 7 (class IV), mu or sigma polypeptide
70385	Spdl1	-1.25	2.12E-02	spindle apparatus coiled-coil protein 1
213006	Mfsd4	-1.27	3.44E-02	major facilitator superfamily domain containing 4
244329	Mcph1	-1.27	4.33E-02	microcephaly, primary autosomal recessive 1
105083	Pelo	-1.28	3.79E-02	pelota homolog (Drosophila)
67390	Rnmtl1	-1.29	2.73E-02	RNA methyltransferase like 1
12971	Crym	-1.31	2.13E-02	crystallin, mu
52653	Nudcd2	-1.33	4.82E-02	NudC domain containing 2
74096	Hvcn1	-1.34	2.86E-02	hydrogen voltage-gated channel 1
242519	Ifna12	-1.37	1.97E-02	interferon alpha 12
17300	Foxc1	-1.38	2.29E-02	forkhead box C1
18782	Pla2g2d	-1.53	2.52E-02	phospholipase A2, group IID
114332	Lyve1	-1.68	1.01E-02	lymphatic vessel endothelial hyaluronan receptor 1
276891	Timd4	-1.74	7.08E-04	T cell immunoglobulin and mucin domain containing 4
14276	Folr2	-1.82	1.65E-02	folate receptor 2 (fetal)
